# Interaction of Aromatic Amino Acids with Metal Complexes of Tetrakis-(4-Sulfonatophenyl)Porphyrin

**DOI:** 10.3390/molecules29020472

**Published:** 2024-01-18

**Authors:** Roberto Zagami, Maria Angela Castriciano, Mariachiara Trapani, Andrea Romeo, Luigi Monsù Scolaro

**Affiliations:** 1 Dipartimento di Scienze Chimiche, Biologiche, Farmaceutiche ed Ambientali, University of Messina, V. le F. Stagno D’Alcontres, 31, 98166 Messina, Italy; rzagami@unime.it (R.Z.); mcastriciano@unime.it (M.A.C.); andrea.romeo@unime.it (A.R.); 2 CNR—ISMN Istituto per lo Studio dei Materiali Nanostrutturati c/o Dipartimento di Scienze Chimiche, Biologiche, Farmaceutiche ed Ambientali, University of Messina, V. le F. Stagno D’Alcontres, 31, 98166 Messina, Italy; mariachiara.trapani@cnr.it

**Keywords:** porphyrins, amino acids, supramolecular adducts, hydrophobicity

## Abstract

The interaction of a series of metal derivatives of 5, 10, 15, 20-*tetrakis*(4-sulfonato-phenyl)porphyrin (MTPPS_4_, M = Cu(II), Pt(II), Ni(II), Zn(II) and Co(II)), including the metal free porphyrin (TPPS_4_), with the aromatic amino acids L-tryptophan (L-Trp), L-and D-phenylalanine (L-and D-Phe) and L-histidine (L-His) have been investigated through UV/Vis spectroscopy. The amino acid L-serine (L-Ser) has been included as reference compound. The spectroscopic changes induced by adding the amino acids have been exploited to evaluate the extent of interaction between the molecular components in the supramolecular adducts. The binding constants have been estimated for most of the investigated systems, assuming a simple 1:1 equilibrium. The bathochromic shifts of the B-bands, the extent of hypochromicity and the binding constants have been analyzed through two chemical descriptors. All the data point to the important role played by the steric hindrance introduced by axial ligands coordinated to the metal ions and to the degree of hydrophobicity and size of the aromatic moiety in the amino acids.

## 1. Introduction

Chirality in supramolecular chemistry is a highly investigated topic [1,2,3]. From a fundamental point of view, it is important to understand the mechanism of chirality transfer or propagation from a chiral monomeric building block to a complex supramolecular architecture [1,4]. More intriguing is the case when chirality occurs upon supramolecular assembling processes starting from achiral building blocks, in the presence of a proper chiral bias [3,5]. Many reports in the literature have pointed out the possibility of controlling the handedness of the final assemblies through chiral chemical [1,6,7,8,9,10,11] or physical stimuli [12,13,14,15,16,17,18,19,20]. In such a context, porphyrins are good model systems to access a variety of supramolecular aggregates. These compounds possess a quite large aromatic region that can lead to stacking interactions among neighboring porphyrins. Moreover, they are easily tunable by peripheral chemical modifications, introducing further side groups with different molecular recognition ability [21,22,23,24,25]. In addition, due to the presence of four central nitrogen atoms, these macrocycles are able to act as ligands for a large number of metal ions, thus exhibiting a rich coordination chemistry [26,27,28]. The size and extent of electronic conjugation of the aromatic core are responsible for their characteristic absorption in the visible range of the UV/Vis spectrum, which is dominated by a very intense B-band in the higher-energy portion accompanied by a variable number of weaker and lower-energy Q-bands [26,27,29]. Because the electronic spectra of porphyrins are very sensitive to slight changes in the microenvironment, their aggregation state or degree of interaction with other species can be easily accessed through modification of the energy and intensity of these bands. In the plethora of aggregated porphyrins, J-aggregates represent an interesting class due to their peculiar electronic features. According to Kasha [30], a lateral geometric arrangement of the chromophores determines a shift of the bands to lower energy, leading to a characteristic red-shifted band. Furthermore, motional narrowing is responsible for the sharpening of the so called J-band [31]. Meso-phenyl substituted porphyrins bearing a variable number of sulphonato groups in para position on the ring are able to self-assemble to form these aggregated species [13,21,32,33]. In particular, the tetra-anionic 5, 10, 15, 20-*tetrakis*(4-sulfonatophenyl)porphyrin (TPPS_4_) has been extensively investigated [34,35,36,37,38,39]. Aggregation can be easily triggered by lowering the pH and increasing the ionic strength, or in the presence of a variety of cationic species [40,41,42,43]. The main species—due to the initial protonation of the nitrogen core and eventually of a couple of sulphonato groups—are able to self-organize through an interplay of non-covalent interactions, including electrostatic, hydrogen-bonding and π-stacking contacts. The resulting nanoaggregates exhibit a variety of architectures [13,32], among which nanotubes have been widely characterized [39]. Chirality can be expressed in these latter ones when they are prepared in the presence of chiral stimuli [40,44,45,46,47,48,49,50,51,52,53,54] or even by spontaneous symmetry breaking [55,56,57]. Kinetic investigations have pointed out that the mechanism of growth is based on an initial nucleation stage that leads to the rate-determining formation of a trimer or tetramer of porphyrin. These latter species are the starting seeds of the auto-catalytic pathway for the eventual J-aggregates [41]. Therefore, all the experimental evidence suggests that the factor influencing the rates, i.e., counter-anions [58] and chiral inducers, seems to be operative at this level. Anyway, many reports in the literature deal with the interaction of simple amino acids with porphyrin receptors. Induction of chirality has been observed in a series of properly functionalized porphyrins, when the contact with the guest amino acid is effective [52,59,60,61,62,63,64,65,66]. In this context, it is important to understand whether the TPPS_4_ porphyrin, under conditions disfavoring the formation of J-aggregates, is able at monomeric level to form any kind of supramolecular species with a simple amino acid. Considering that electrostatics, hydrogen bonding and solvophobic interactions largely contribute to the stabilization of such species, we expect that the presence of an aromatic moiety on the side chain of the amino acid could provide a further driving force to the overall binding. Here we report an investigation on the interaction of some aromatic amino acids with a series of divalent metal ion derivatives of TPPS_4_, including the metal free porphyrin. In particular, Cu(II), Pt(II), Ni(II), Zn(II) and Co(II) have been selected to study the progressive impact of the coordination geometry imposed by the metal ion. Besides hampering the formation of J-aggregates, we anticipate that the absence/presence of axial ligands bound to the metal and generating steric hindrance on the porphyrin plane has a deep effect on the degree of interaction, as measured by the modification of the electronic absorption properties and the binding constants. The results are in line with the extent of hydrophobicity and the size of the aromatic region of the amino acids.

## 2. Results

**Scheme 1:**
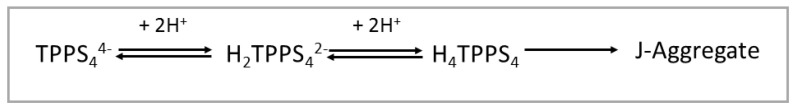
Protonation state for TPPS_4_ porphyrin and formation of J-aggregates as function of pH.

TPPS_4_ porphyrin leads to various species with different protonation degrees and overall charges depending on the acidity of the medium [35]. Under neutral pH conditions, this compound has an overall −4 charge (TPPS_4_^4−^), since all the sulphonato groups are ionized. Since the pK_a_ for the protonation of the nitrogen atoms of the macrocycle is 4.9 [67], below this value of pH the main prevailing species is a di-anion (H_2_TPPS_4_^2−^). At pH lower than 3, a neutral zwitter-ion (H_4_TPPS_4_) forms due to protonation of two sulphonato groups [35]. This latter species can eventually self-organize into J-aggregates, under the proper conditions of pH, ionic strength and presence of cationic species (Scheme 1) [35].

**Scheme 2:**
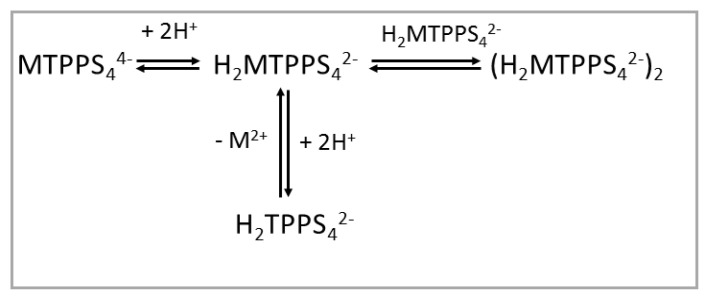
Protonation state for MTPPS_4_^4−^ porphyrins as a function of pH.

When the TPPS_4_ porphyrin is bound to divalent metal ions forming MTPPS_4_^4−^ coordination complexes, the protonation behavior is obviously different, as the porphyrin core is blocked by the presence of the metal ion and the nitrogen atoms are not available for binding hydrogen ions. The effect of increasing the proton concentration is (i) protonation of at least two of the sulphonato groups, thus leading to a H_2_MTPPS_4_^2−^ species, and (ii) that depending on the lability of the metal ion, demetallation can take place (e.g., for the labile ZnTPPS_4_^4−^) [42], with formation of the diacid species H_2_TPPS_4_^2−^. In the case of CuTPPS_4_^4−^, under quite acidic conditions, demetallation is still slow, and dimerization can occur (Scheme 2) [68]. Considering the higher inertness of Ni(II), Co(II), Cu(II) and Pt(II) with respect to the acid solvolysis reaction, at rather low porphyrin concentration and mild acidic conditions, all the relative metal derivatives do not undergo demetallation. 

The conditions used in our experiments (pH = 4 in acetate buffer and porphyrin concentration 1.5 µM) ensure that all the porphyrin metal complexes are actually present as tetra-anions and that the metal free ligand is in its di-anionic H_2_TPPS_4_^2−^ form. As far as the amino acids are concerned, the values of their isoelectric points are higher than 4 (Trp, 5.89; Phe, 5.48; His, 7.59; Ser, 5.68). This condition implies that the species in solution is the mono-cation, histidine being the only exception due to the double protonation of the α-amino group and the imidazole nitrogen on the side chain. 

**Figure 1:**
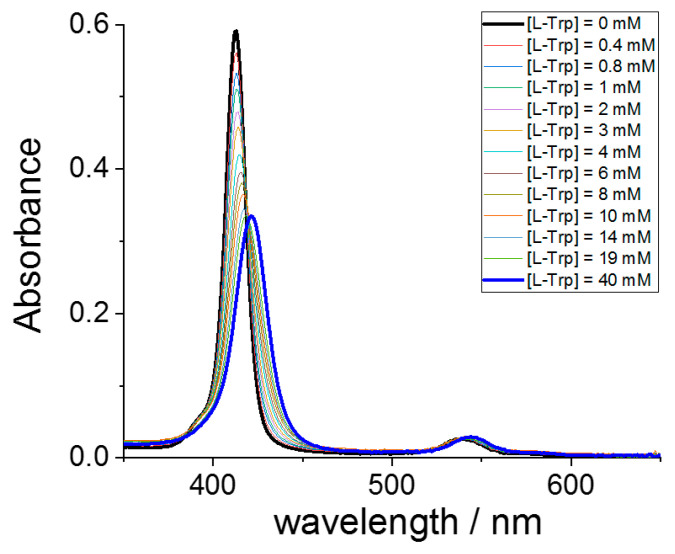
UV/Vis absorbance spectral changes for the progressive titration of CuTPPS_4_^4−^ (black line) with L-Trp up to a concentration of 40 mM (blue line). Experimental conditions: [CuTPPS_4_^4−^] = 1.5 μM; [L-Trp] = 0–40 mM; acetate buffer 100 mM pH = 4, T = 298 K, cell path length 1 cm.

To study the interaction between the two components, we have performed a series of UV/Vis titrations by adding the various amino acids to the selected metal derivatives of TPPS_4_, including the metal free ligand. As an example, Figure 1 shows a typical spectral change observed for the interaction of CuTPPS_4_^4−^ and L-Trp. This porphyrin exhibits a strong B-band at 413 nm, accompanied by two weaker Q-bands at lower energy. The progressive addition of L-Trp leads to a bathochromic shift of the B-band that eventually moves to 421 nm (Δλ = +8 nm), accompanied by a consistent hypochromicity. Two distinct isosbestic points can be detected at 419 and 538 nm. Under these experimental conditions, J-aggregates do not form, as is possible to observe by the absence of the characteristic J-band at 490 nm.

The circular dichroism spectrum (CD) measured on the solution containing the higher L-Trp concentration shows no induced bands in the region of the porphyrin absorption, suggesting that tryptophan is not able to transfer the chiral information to the supramolecular adduct. 

Analogous titration experiments have been performed for the other amino acids and porphyrins. In all these cases, the extent of the observed bathochromic shift and the hypochromicity are lower. In particular, in the case of CuTPPS_4_^4−^ with L-His and L-Ser, and CoTPPS_4_^4−^ with L-Trp, no shift of the B-band has been detected. The diacid porphyrin H_2_TPPS_4_^2−^ does not evidence any red shift, even if its Soret band undergoes a discrete reduction in intensity. To evaluate the extent of hypochromicity, we have applied the following relationship: H% = 100 × (Abs_max_ − Abs_min_)/Abs_max_, where H% is the percentage of hypochromicity, Abs_max_ is the maximum absorbance value for the initial concentration of porphyrin in the absence of amino acid at the B-band and Abs_min_ is the final absorbance value for the adduct with the amino acid. The observation of bathochromic shifts in the B-bands, in association with hypochromicity, points to the electronic interaction between the porphyrin (P) and the amino acids (AA), thus leading to the supramolecular species P@AA, in agreement with similar findings in the literature. The amount of red shift is generally related to the stacking interaction of the aromatic portion of the amino acid and the porphyrin macrocycle. All the data relative to the bathochromic shifts and the hypochromicity (H%) are collected in Table 1. For all the final adducts, the CD spectra do not display any induced signal in the B-band region. This observation is different from what is normally observed for the aggregated form of the free ligand, since chirality is easily induced by amino acids and other chiral species in J-aggregates of the parent TPPS_4_ porphyrin [52]. The occurrence of a chiral arrangement of the porphyrin building blocks in the growing supramolecular structure at the nano- and mesoscopic levels is proved by the appearance of strong CD bands, whose sign is controlled by the handedness of the added chiral templating reagents. Our experimental evidence suggests that the chiral bias should play a role at the level of the rate-determining step of the J-aggregates formation. Kinetic investigations have already pointed out that, after a preliminary nucleation period, the auto-catalytic pathway leading to the final aggregated species is controlled by the formation of trimers or tetramers [35,52]. Consequently, the formation of a chiral assembly should involve such species and not the simple monomeric porphyrin.

**Figure 2:**
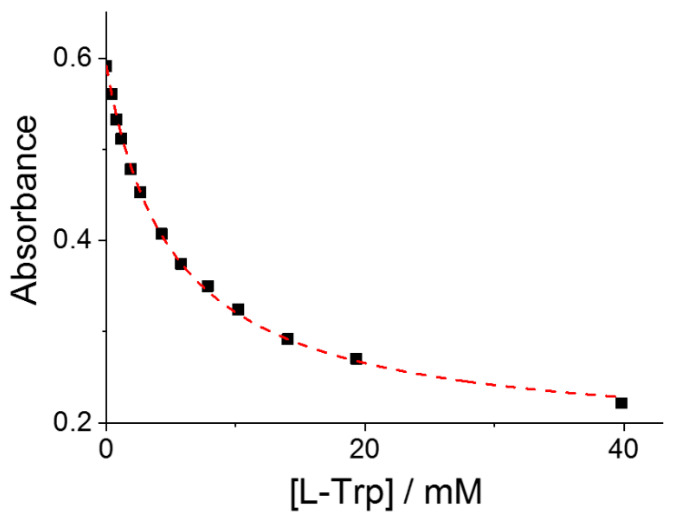
Absorbance changes at the B-band of CuTPPS_4_^4−^ as a function of the total concentration of added L-Trp. The red curve is the best fitting of the experimental data to Equation (6) (see Section 3): *K_eq_* = 212 ± 5; ε_P@AA_ = (1.14 ± 0.03) × 10^5^ M^−1^cm^−1^; R^2^ = 0.9989. Experimental conditions: [CuTPPS_4_^4−^] = 1.5 μM; [L-Trp] = 0–40 mM; acetate buffer 100 mM, pH = 4; T = 298 K; cell path length 1 cm.

The binding constants (*K_eq_*) for the formation of the supramolecular adduct P@AA have been evaluated assuming a simple 1:1 equilibrium between the porphyrin and the selected amino acid. Consequently, the absorbance data collected at the wavelength corresponding to the maximum spectral change, usually at the B-band of the starting porphyrin, have been fitted to Equation (6) as a function of the total amino acid concentration (see Section 3). Figure 2 shows a typical curve fitting obtained for the titration of CuTPPS_4_^4−^ with L-Trp. This procedure allowed us to obtain the values of *K_eq_* and the molar extinction coefficient, ε_P@AA_, for the various adducts P@AA, except for the cases where the spectral changes were negligible (CuTPPS_4_^4−^ with L-Ser and CoTPPS_4_^4−^ with L-Trp). All these data are collected in Table 1 (Figures S1–S11). 

The experimental findings allow us to correlate the degree of interaction in the various supramolecular adducts and the structural features of both porphyrins and amino acids. As previously mentioned, the choice of the metal ions in MTPPS_4_^4−^ has been oriented to the possibility of modulating the steric properties of the macrocycle. Whereas Pt(II) and Cu(II) usually form tetra-coordinated complexes with a square planar geometry, Ni(II) has the ability to vary the coordination number spanning from 4 to 6 [69], Zn(II) is penta-coordinated and Co(II) in most cases binds its ligands, affording hexa-coordinated complexes. In our experimental pH conditions, the axial ligand should be water in all the metal complexes with a coordination number higher than 4. Therefore, we can organize the various metal complexes MTPPS_4_^4−^ in increasing order of steric hindrance, according to the following series: CuTPPS_4_^4−^ ~ PtTPPS_4_^4−^ ~ NiTPPS_4_^4−^ < ZnTPPS_4_^4−^ < CoTPPS_4_^4−^. The metal free ligand H_2_TPPS_4_^2−^ used for the sake of comparison is not planar, but in a saddled conformation, i.e., the pyrrole rings are alternatively oriented up and down with respect to the mean plane of the macrocycle. Considering that tryptophan is the amino acid that binds more tightly in comparison with the others, we decided to select this compound as a reference point. 

**Figure 3:**
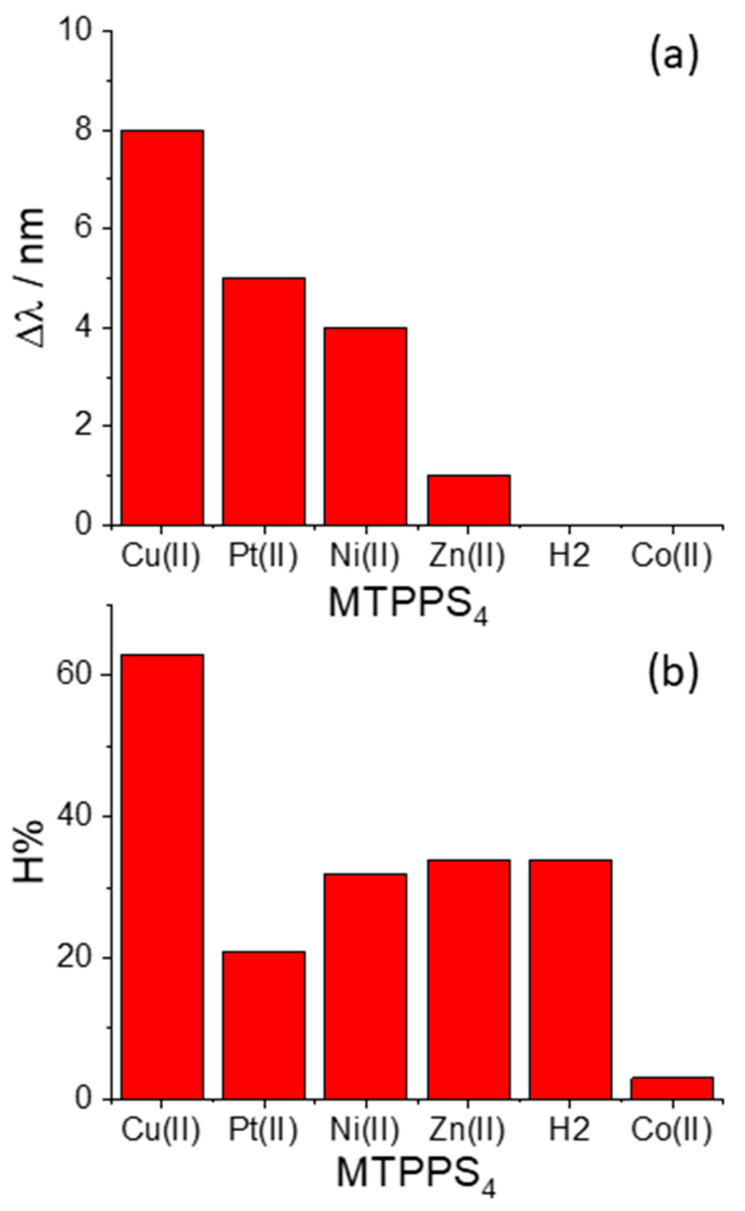
Plot of the values for (**a**) the bathochromic shifts (Δλ) and (**b**) the hypochromicity degree (H%) for the various metal derivatives MTPPS_4_^4−^ with the amino acid L-Trp.

**Figure 4:**
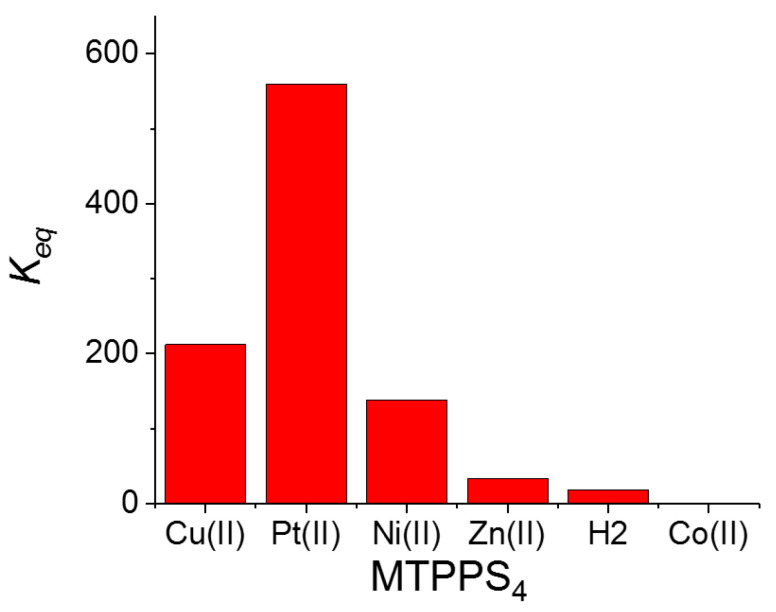
Plot of the values for the equilibrium binding constants *K_eq_* for the various metal derivatives MTPPS_4_^4−^ with the amino acid L-Trp.

The pattern of behavior that the bathochromic shifts (Δλ) and the hypochromicity (H%) exhibit in the various species MTPPS_4_^4−^@L-Trp is displayed in Figure 3a,b, respectively. An inspection of the data reveals that the Δλ values indicate the following sequence: CuTPPS_4_^4−^ > PtTPPS_4_^4−^ > NiTPPS_4_^4−^ >> ZnTPPS_4_^4−^ >> H_2_TPPS_4_^2−^ ~ CoTPPS_4_^4−^. This result is in line with (i) the absence of axial ligands in the metal complexes containing Cu(II), Pt(II) and Ni(II), and (ii) an axial ligand in the case of the Zn(II) porphyrin and (iii) two axial ligands for Co(II) porphyrin. Therefore, the steric hindrance caused by two axial ligands coordinated above and below the porphyrin plane seems to play a role as important as the deformation of the macrocycle core induced by the protonation of the nitrogen atoms. When considering the hypochromicity, the data do not exhibit any relevant trend related to the number of coordinated solvent molecules, apart from the steep decrease observed for CoTPPS_4_^4−^ (Figure 3b). The values of the binding constants *K_eq_* are reported in Figure 4 and follow the trend: PtTPPS_4_^4−^ >> CuTPPS_4_^4−^ > NiTPPS_4_^4−^ >> ZnTPPS_4_^4−^ > H_2_TPPS_4_^2−^ > CoTPPS_4_^4−^, which is in agreement with the gradual decrease of the binding force on increasing the number of the axial ligands on the porphyrin plane.

**Figure 5:**
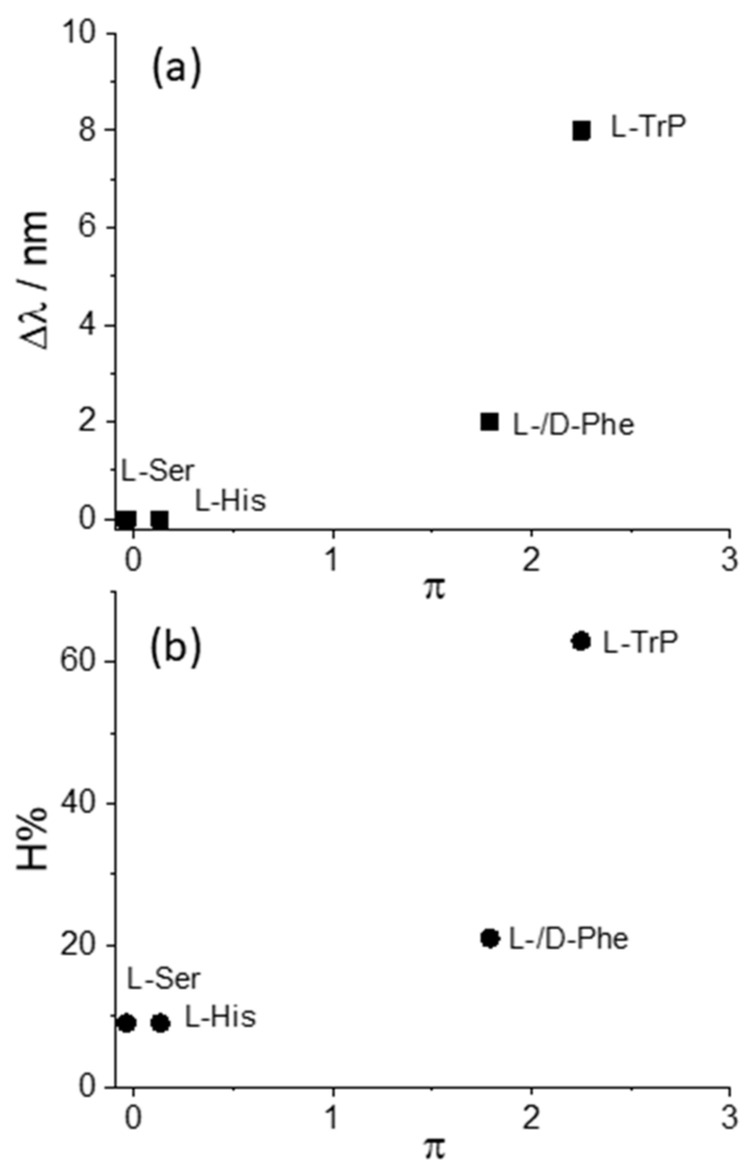
Plot of the values for the (**a**) bathochromic shift values, Δλ, and (**b**) the hypochromicity extent, H%, as functions of the hydrophobicity parameter, π, for CuTPPS_4_^4−^ interacting with the various amino acids.

**Figure 6:**
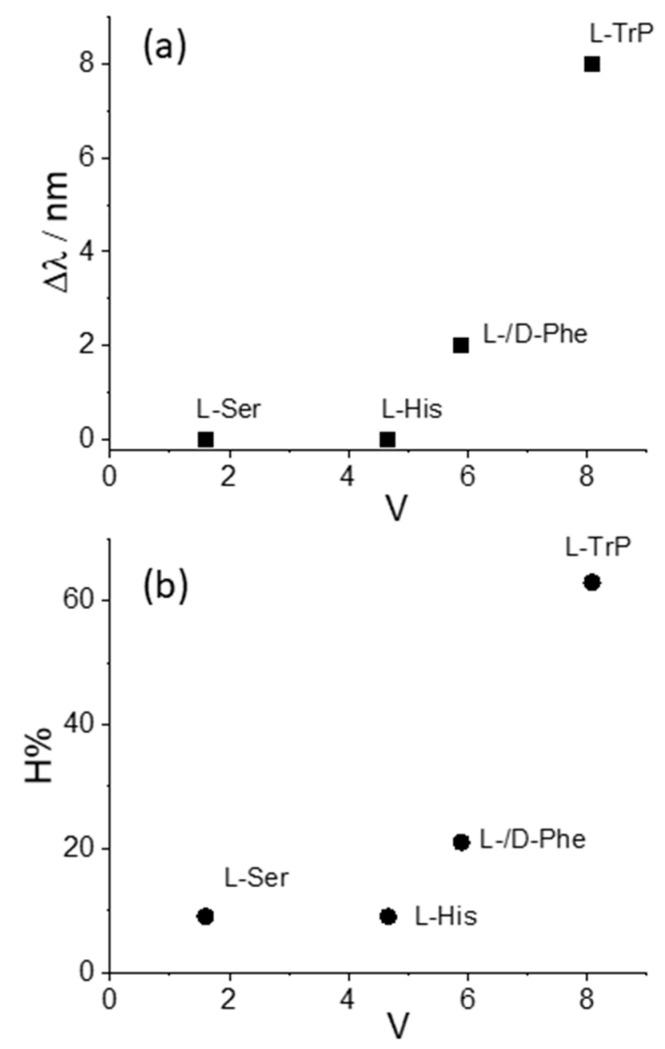
Plot of the values for the (**a**) bathochromic shift values, Δλ, and (**b**) the hypochromicity extent, H%, as functions of the normalized van der Waals volume, V, for CuTPPS_4_^4−^ interacting with the various amino acids.

We decided to take advantage of some chemical descriptors, commonly used to analyze properties of the amino acids in proteins, to find a rationale in our data [70]. Taking into account the Cu derivative revealed to be the most sensible to interact with L-Trp, we adopted (i) hydrophobicity, π, defined as *log P_(amino acid)_* − *log P_(glycine)_*, where *log P* is the partition coefficient of the specific amino acid in 1-octanol/water mixture, and (ii) the normalized van der Waals volume, V, of the side chain, calculated by the equation V = [V_(*side chain*)_ − V_(*H*)_]/V_(*CH*2)_ [70]. This parameter is 0 for glycine and 1 for alanine. The values of these two descriptors for the amino acids investigated in our study decrease in the order Trp > Phe > His > Ser, as expected based on the relative molecular size. Figure 5 and Figure 6 report the plots of Δλ and H% as functions of the hydrophobicity π and normalized van der Waals volume V, respectively, for the various amino acids. A monotonically increasing trend is evident in all these graphics, following the increase of the size of the aromatic side chain and the hydrophobicity of the amino acids. 

**Figure 7:**
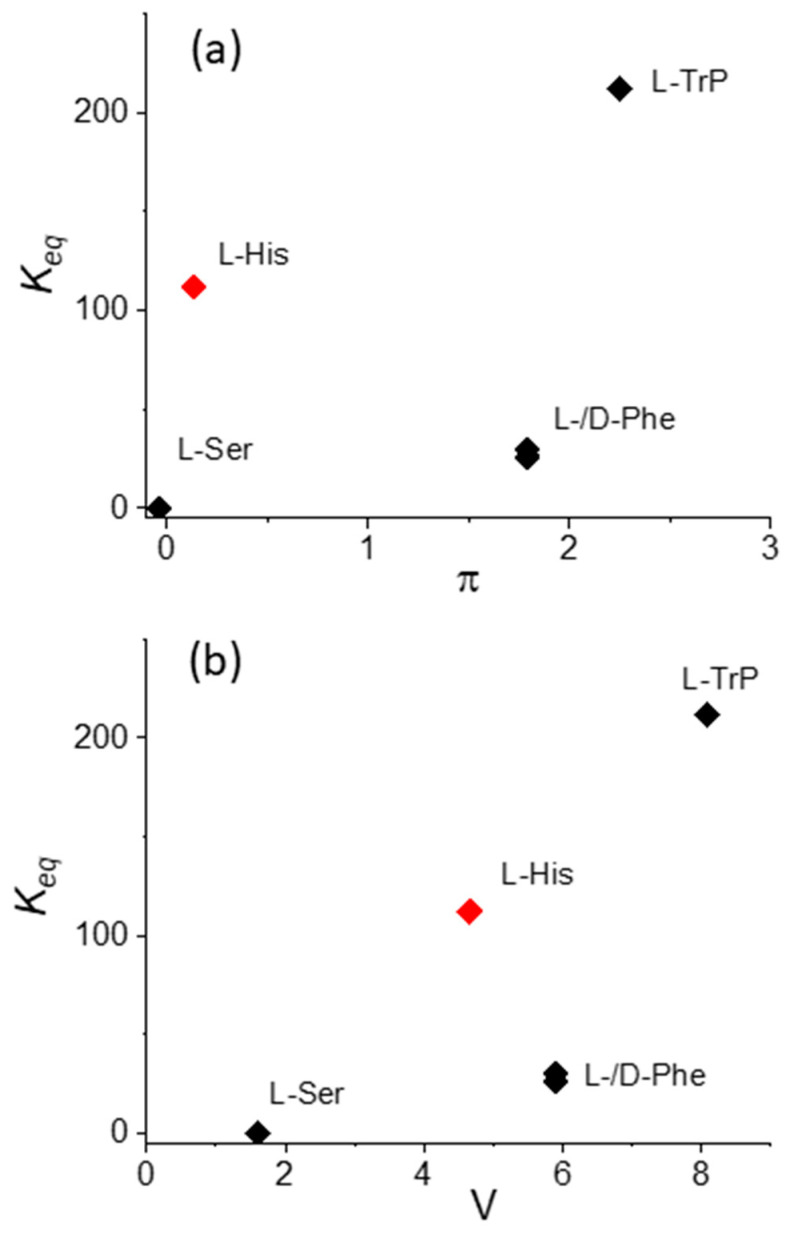
Plot of the values for the equilibrium binding constants *K_eq_* versus (**a**) hydrophobicity, π, and (**b**) the normalized van der Waals volume, V, for CuTPPS_4_^4−^ interacting with the various amino acids.

If the L-His data is excluded, Figure 7 shows that also in the case of the binding constants *K_eq_*, a monotonically increasing trend, with both π and V increasing, is evident. The anomalous position of the value for L-His should be explained by (i) the presence of the donor nitrogen atom on the imidazole residue, which is able to coordinate or interact with the Cu(II) metal center in the macrocycle, and (ii) the overall charge on this specific amino acid, which, as pointed out above, at pH 4 is +2, due to the protonation of both the α-amino group and the imidazole nitrogen on the side chain. It is interesting to observe that, as expected, an inspection of the data relative to the enantiomers L- and D-Phe does not evidence any difference in terms of electronic interactions and binding constant values. 

**Figure 8:**
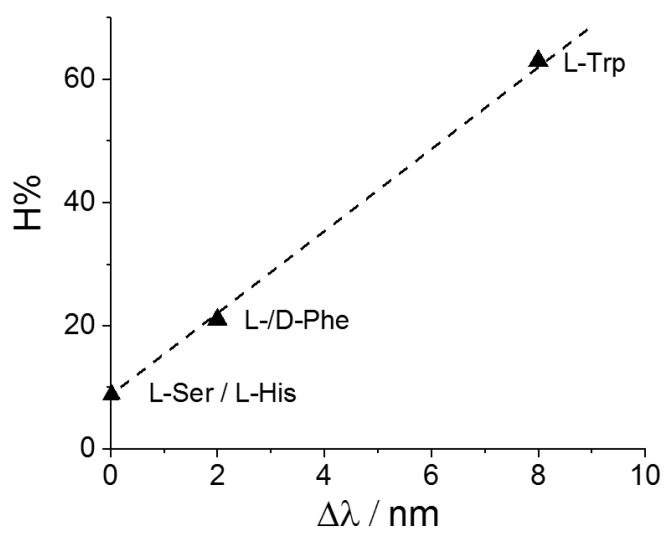
Plot of the values for the hypochromicity extent, H%, as a function of the observed bathochromic shift values, Δλ, for CuTPPS_4_^4−^ interacting with the various amino acids.

Figure 8 displays a plot of the hypochromicity extent H% versus the bathochromic shift Δλ for the adducts CuTPPS_4_^4−^@AA. It is interesting to observe that these two spectroscopic parameters are linearly dependent, at least for this specific metal derivative. This evidence suggests that a common mechanism is operative and related to the extent of perturbation exerted by the aromatic moiety of the amino acid on the electronic distribution of the porphyrin core, reflected both in the hypochromicity and the bathochromic shift of the band. On the other hand, no clear correlation has been observed when the different MTPPS_4_^4−^@L-Trp adducts are considered. All the spectroscopic data suggest that electrostatics and stacking interactions are the main driving forces in stabilizing these supramolecular species. 

## 3. Materials and Methods

### 3.1. Materials

5, 10, 15, 20-*tetrakis*(4-sulfonatophenyl)porphyrin (TPPS_4_), as sodium salt, was received from Aldrich (Milan, Italy). All the amino acids (L-tryptophan, L-phenylalanine, D-phenylalanine, L-histidine and L-serine), sodium acetate and acetic acid were of the highest commercial grade available and were used as received, without further purification, from Sigma-Aldrich (Milan, Italy). All the aqueous solutions were prepared in high-purity doubly distilled water (HPLC grade, Fluka, Milan, Italy). Stock solutions of the various porphyrins (100–200 μM) were freshly prepared and stored in the dark to avoid photo-damage. The concentration of the samples used in the experiments was calculated by UV/Vis absorption spectroscopy using the molar extinction coefficients at the B-band (TPPS_4_: 5.33 × 10^5^ M^−1^ cm^−1^, λ = 414 nm; NiTPPS_4_: 2.7 × 10^5^ M^−1^ cm^−1^, λ = 409 nm; ZnTPPS_4_: 6.8 × 10^5^ M^−1^ cm^−1^, λ = 422 nm; CuTPPS_4_: 4.2 × 10^5^ M^−1^ cm^−1^, λ = 413 nm; CoTPPS_4_: 2.7 × 10^5^ M^−1^ cm^−1^, λ = 425 nm; PtTPPS_4_: 3.7 × 10^5^ M^−1^ cm^−1^, λ = 397 nm). The amino acid solutions for the titration experiments have been prepared by solubilizing these compounds in 100 mM acetate buffer at pH = 4.

### 3.2. Methods

UV/Vis extinction spectra were collected on an Agilent 8453 diode array spectrophotometer. In order to prevent the light-induced degradation of the porphyrin solutions during the experiments, we used a UV filter (Hoya glass type UV-34, cut-off: 340 nm) set between the lamp and the samples. Temperature was controlled at 298 K by an external water-circulating bath. CD spectra were recorded on a Jasco J-710 spectropolarimeter, Jasco Europe.

Titration experiments were performed by collecting UV/Vis spectra on solutions contained in quartz Hellma cells placed in the thermostatic holder of the spectrophotometer. In a general procedure, small aliquots of a stock solution containing the selected AA (40 mM) and porphyrin (1.5 µM) were progressively added to 2 mL of a prediluted MTPPS_4_^4−^ 1.5 μM solution in a 1 cm pathlength cell. The absorbance values (Abs) have been collected at the wavelength of maximum change, generally at the B-band of the initial MTPPS_4_^4−^ species. In order to evaluate the equilibrium binding constants, we have considered a simple 1:1 equilibrium between porphyrin and AA ligand:

P + AA ⇄ P@AA

with an equilibrium binding constant

*K_eq_* = [P@AA]/([P] × [AA]) (1)

Considering the mass balance of porphyrin,

[P]_T_ = [P] + [P@AA] (2)

([P] and [P]_T_ are the free and total porphyrin concentration, respectively, while [P@AA] is the supramolecular adduct concentration) and AA ligand,

[<=>AA]_T_ = [AA] + [P@AA] (3)

([AA] and [AA]_T_ are the free and total AA concentration, respectively), the following equation can be derived:

*K_eq_*[P]^2^ + (1 + *K_eq_*([AA]_T_ − [P]_T_)) [P] − [P]_T_ = 0 (4)

from which the value of the free porphyrin concentration [P] can be obtained:

[P] = −(1 + *K_eq_* ([AA]_T_ − [P]_T_)) + ((1 + *K_eq_* ([AA]_T_ − [P]_T_))^2^ + 4 *K_eq_* [P]_T_)^1/2^}/(2 *K_eq_*) (5)

The absorbance data have been analyzed through a non-linear best fitting procedure of the absorbance data at the B-band maximum as a function of the total AA concentration, according to the following equation:

Abs = ε_P_ [P] + ε_P@AA_ ([P]_T_ − [P]) (6)

where ε_P_ and ε_P@AA_ are the molar extinction coefficients of the metal derivatives and the supramolecular adducts with the AA, respectively.

## 4. Conclusions

Amino acids bearing an aromatic residue on the side chain are able to interact with TPPS_4_ and its metal complexes MTPPS_4_ through a combination of weak interactions. Among these forces, electrostatics play a very important role as, under the experimental conditions adopted in our experiments, the AA are in cationic form and the MTPPS_4_ derivatives are tetra-anions, while TPPS_4_ is a di-anion. In addition, hydrogen bonding and hydrophobic interactions through π-stacking of the aromatic moieties are present on both the interacting species in the supramolecular adducts. Experimental evidence based on the electronic perturbation and the binding equilibrium constant values indicates that the absence of steric hindrance above and below the porphyrin plane favors the interaction with AA. This condition is ensured by transition metal ions exhibiting square planar coordination geometry (i.e., Cu(II), Pt(II) and Ni(II)). The introduction of axial ligands leads to a steep decrease in the extent of interaction. In the case of Co(II)TPPS_4_, the presence of two solvent molecules bound to the metal center reduces even further the possibility of π-stacking contacts. The role of the size of the aromatic region of the AA finds a rationale considering descriptor parameters, i.e., the hydrophobicity and the normalized van der Waals volume, that have been developed in the literature to model the behavior of these compounds in proteins and other biological systems.

The absence of any detectable induced CD spectra of the adducts between the metallo-porphyrins and the AA points to a role of these latter compounds in the aggregation pathway to chiral J-aggregates only at the level of the rate-determining step, when trimers or tetramers must be formed.

## Figures and Tables

**Table 1:** Bathochromic shift of the B-band (Δλ), hypochromicity (H%), equilibrium binding constants (*K_eq_*) and molar extinction coefficients (ε_P@AA_) for the P@AA supramolecular adducts ^a^.

MTPPS_4_^4−^	AA	Δλ (nm)	H%	*K_eq_*	10^−5^ εP@AA (M^−1^ cm^−1^)
Cu(II)	L-Trp	8	63	212 ± 5	1.14 ± 0.03
Cu(II)	L-Phe	2	21	26 ± 2	1.93 ± 0.09
Cu(II)	D-Phe	2	21	30 ± 3	2.14 ± 0.01
Cu(II)	L-His	-	9	112 ± 14	3.57 ± 0.03
Cu(II)	L-Ser	-	9	-	-
Pt(II)	L-Trp	5	21	560 ± 66	3.11 ± 0.02
Ni(II)	L-Trp	4	32	138 ± 13	1.69 ± 0.04
Ni(II)	L-Phe	2	11	73 ± 22	2.34 ± 0.05
Ni(II)	D-Phe	2	10	96 ± 17	2.34 ± 0.03
Zn(II)	L-Trp	1	34	33 ± 1	2.65 ± 0.01
Co(II)	L-Trp	-	3	-	-
2H	L-Trp	-	34	18 ± 2	1.03 ± 0.03

^a^ The values of the reported parameters have been obtained in acetate buffer (100 mM) at pH 4 and T = 298 K.

## Data Availability

The data are available upon request.

## Supplementary Materials

The following supporting information can be downloaded at: https://www.mdpi.com/article/10.3390/molecules29020472/s1, Figures S1–S8: UV/Vis absorption spectral changes and absorbance changes at the B-band of MTPPS_4_^4−^ as function of the total concentration of different added aminoacids. Figure S9: UV/Vis absorption spectral changes and absorbance changes at the B-band of H_2_TPPS_4_^4−^ as function of the total concentration of added L-Trp. Figure S10: UV/Vis spectra of CoTPPS_4_^4−^ with and without L-Trp. Figure S11: UV/Vis spectra of CuTPPS_4_^4−^ with and without L-Ser.
