# Supplementary material for: Interaction of Aromatic Amino Acids with Metal Complexes of Tetrakis-(4-Sulfonatophenyl)Porphyrin
Source: Molecules. 2024 Jan 18;29(2):472. doi: 10.3390/molecules29020472 (PMC10819004; doi:10.3390/molecules29020472)
Supplement: Supplementary file 1 [file molecules-29-00472-s001.zip › molecules-2814504-supplementary.pdf]

Electronic Supporting Information

for

Interaction of Aromatic Aminoacids with Metal Complexes of Tetrakis-(4-sulfonatophenyl)porphyrin

Roberto Zagami,[1] Maria Angela Castriciano,[1] Mariachiara Trapani,[2] Andrea Romeo,[1,2] and Luigi Monsù Scolaro*[1,2]

[1] Dipartimento di Scienze Chimiche, Biologiche, Farmaceutiche ed Ambientali, University of Messina, V.le F. Stagno D'Alcontres, 31 - 98166 Messina, Italy; rzagami@unime.it (R.Z.); maria.castriciano@unime.it (M.C.); anromeo@unime.it (A.R.); lmonsu@unime.it (L.M.S.)
[2] CNR - ISMN Istituto per lo Studio dei Materiali Nanostrutturati c/o Dipartimento di Scienze Chimiche, Biologiche, Farmaceutiche ed Ambientali, University of Messina, V.le F. Stagno D'Alcontres, 31 - 98166 Messina, Italy; mariachiara.trapani@cnr.it (M. T.)

Table of contents

Figure S1. UV/Vis Titration of $CuTPPS_4^{4-}$ with L-Phe.

Figure S2. UV/Vis Titration of $CuTPPS_4^{4-}$ with D-Phe.

Figure S3. UV/Vis Titration of $CuTPPS_4^{4-}$ with L-His.

Figure S4. UV/Vis Titration of $PtTPPS_4^{4-}$ with L-Trp.

Figure S5. UV/Vis Titration of $NiTPPS_4^{4-}$ with L-Trp.

Figure S6. UV/Vis Titration of $NiTPPS_4^{4-}$ with L-Phe.

Figure S7. UV/Vis Titration of $NiTPPS_4^{4-}$ with D-Phe.

Figure S8. UV/Vis Titration of $ZnTPPS_4^{4-}$ with L-Trp.

Figure S9. UV/Vis Titration of $H_2TPPS_4^{4-}$ with L-Trp.

Figure S10. UV/Vis Spectra of $CoTPPS_4^{4-}$ with L-Trp.

Figure S11. UV/Vis Spectra of $CuTPPS_4^{4-}$ with L-Ser.

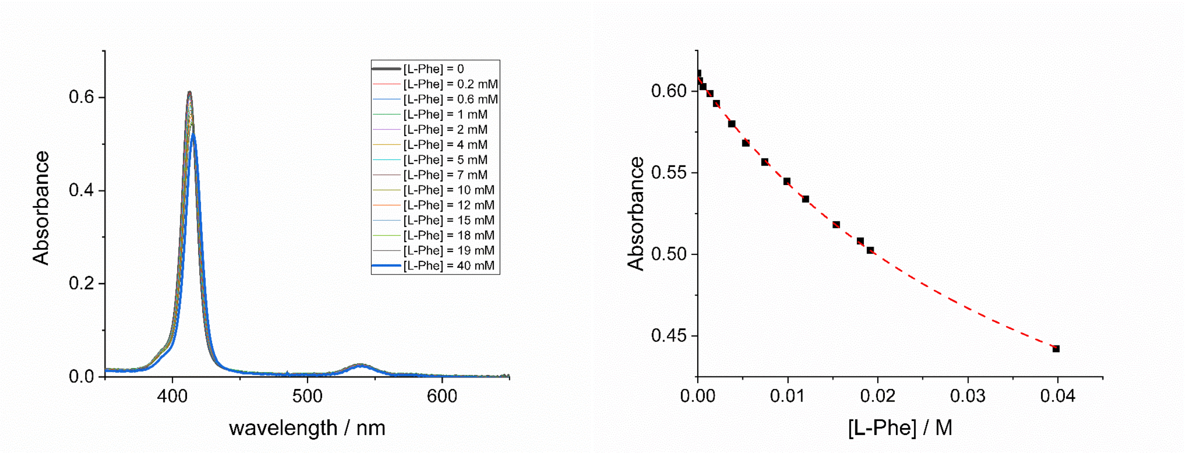

Figure S1. (left) UV/Vis absorption spectral changes for the progressive titration of CuTPPS$_4^{4-}$ (black line) with L-Phe up to a concentration of 40 mM (blue line). **(right)** Absorbance changes at the B-band of CuTPPS$_4^{4-}$ as function of the total concentration of added L-Phe. The red curve is the best fitting of the experimental data to eq. 6 (see Materials and methods). Experimental conditions: [CuTPPS$_4^{4-}$] = 1.5 μM; [L-Phe] = 0 – 40 mM; acetate buffer 100 mM, pH = 4; T = 298 K; cell path length 1 cm.

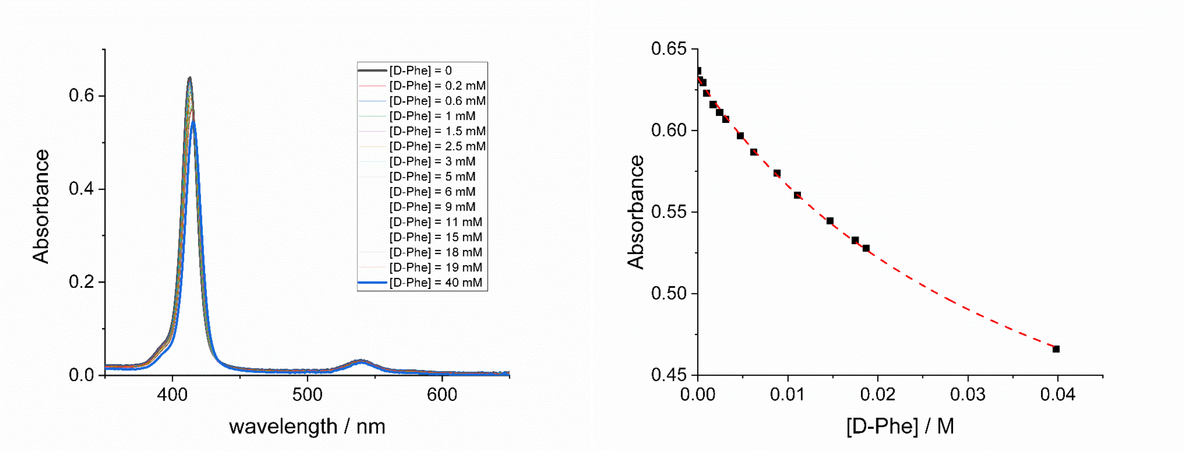

Figure S2. (left) UV/Vis absorption spectral changes for the progressive titration of CuTPPS$_4^{4-}$ (black line) with D-Phe up to a concentration of 40 mM (blue line). **(right)** Absorbance changes at the B-band of CuTPPS$_4^{4-}$ as function of the total concentration of added D-Phe. The red curve is the best fitting of the experimental data to eq. 6 (see Materials and methods). Experimental conditions: [CuTPPS$_4^{4-}$] = 1.5 μM; [D-Phe] = 0 – 40 mM; acetate buffer 100 mM, pH = 4; T = 298 K; cell path length 1 cm.

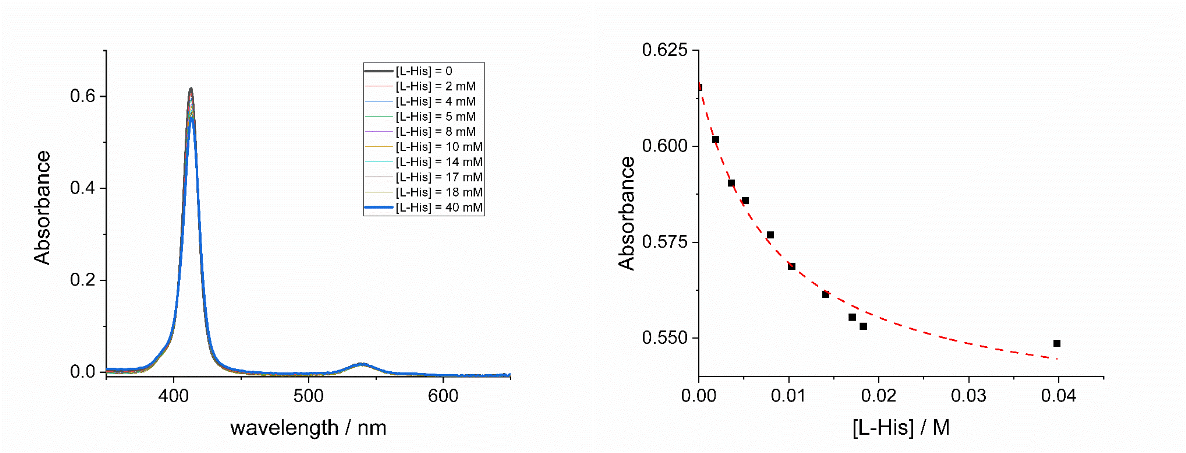

Figure S3. (left) UV/Vis absorption spectral changes for the progressive titration of $CuTPPS_4^{4-}$ (black line) with L-His up to a concentration of 40 mM (blue line). **(right)** Absorbance changes at the B-band of $CuTPPS_4^{4-}$ as function of the total concentration of added L-His. The red curve is the best fitting of the experimental data to eq. 6 (see Materials and methods). Experimental conditions: [$CuTPPS_4^{4-}$] = 1.5 μM; [L-His] = 0 – 40 mM; acetate buffer 100 mM, pH = 4; T = 298 K; cell path length 1 cm.

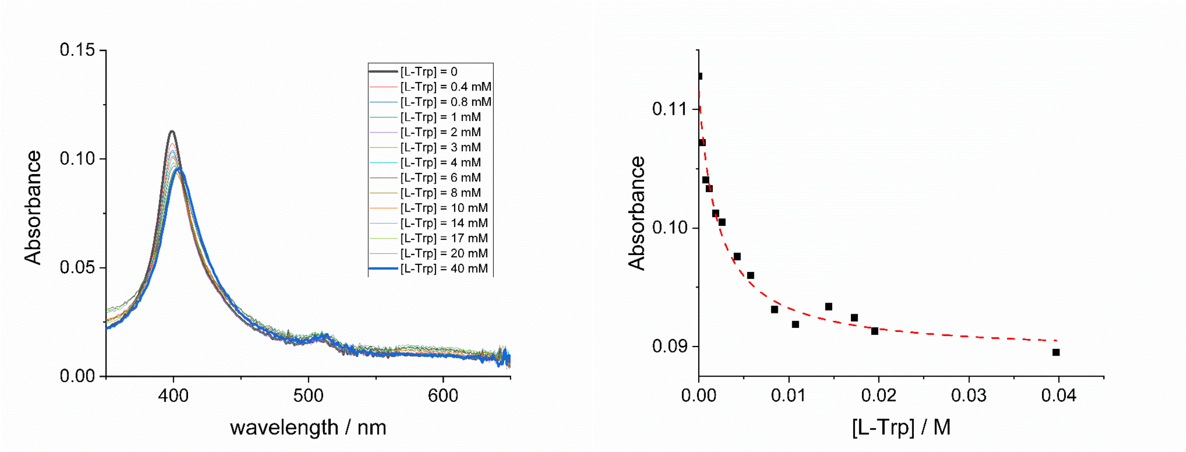

Figure S4. (left) UV/Vis absorption spectral changes for the progressive titration of $PtTPPS_4^{4-}$ (black line) with L-Trp up to a concentration of 40 mM (blue line). **(right)** Absorbance changes at the B-band of $PtTPPS_4^{4-}$ as function of the total concentration of added L-Trp. The red curve is the best fitting of the experimental data to eq. 6 (see Materials and methods). Experimental conditions: [$PtTPPS_4^{4-}$] = 1.5 μM; [L-Trp] = 0 – 40 mM; acetate buffer 100 mM, pH = 4; T = 298 K; cell path length 1 cm.

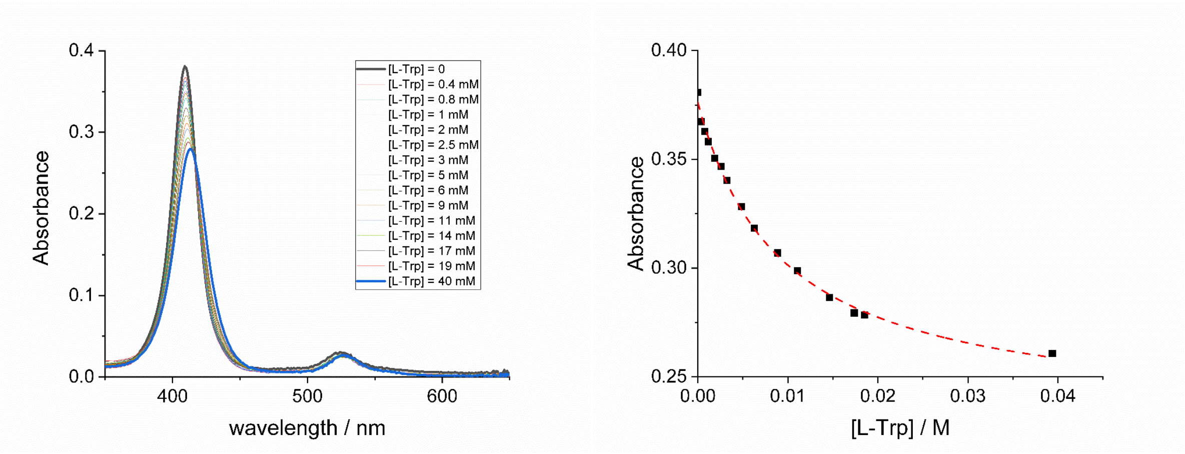

Figure S5. (left) UV/Vis absorption spectral changes for the progressive titration of $NiTPPS_4^{4-}$ (black line) with L-Trp up to a concentration of 40 mM (blue line). **(right)** Absorbance changes at the B-band of $NiTPPS_4^{4-}$ as function of the total concentration of added L-Trp. The red curve is the best fitting of the experimental data to eq. 6 (see Materials and methods). Experimental conditions: [$NiTPPS_4^{4-}$] = 1.5 μM; [L-Trp] = 0 – 40 mM; acetate buffer 100 mM, pH = 4; T = 298 K; cell path length 1 cm.

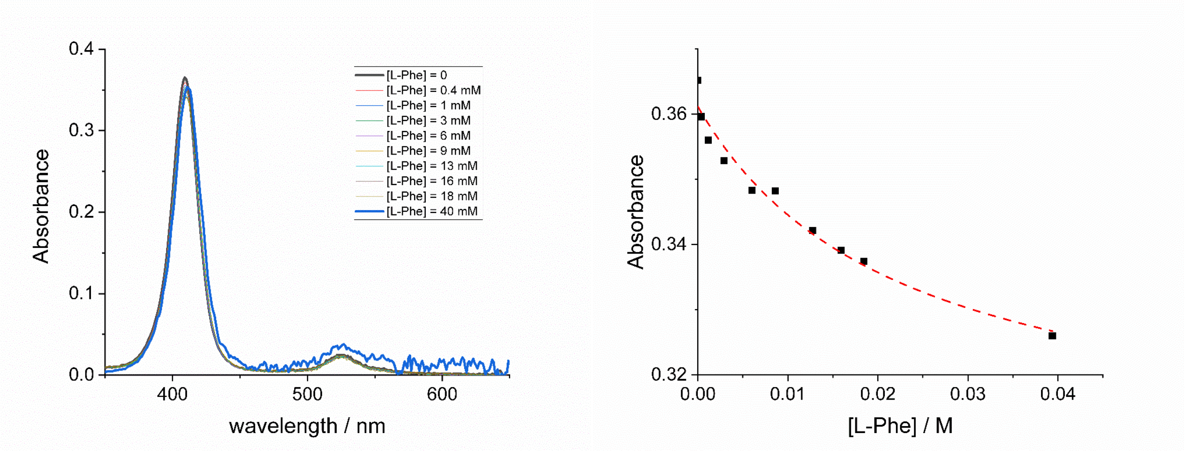

Figure S6. (left) UV/Vis absorption spectral changes for the progressive titration of $NiTPPS_4^{4-}$ (black line) with L-Phe up to a concentration of 40 mM (blue line). **(right)** Absorbance changes at the B-band of $NiTPPS_4^{4-}$ as function of the total concentration of added L-Phe. The red curve is the best fitting of the experimental data to eq. 6 (see Materials and methods). Experimental conditions: [$NiTPPS_4^{4-}$] = 1.5 μM; [L-Phe] = 0 – 40 mM; acetate buffer 100 mM, pH = 4; T = 298 K; cell path length 1 cm.

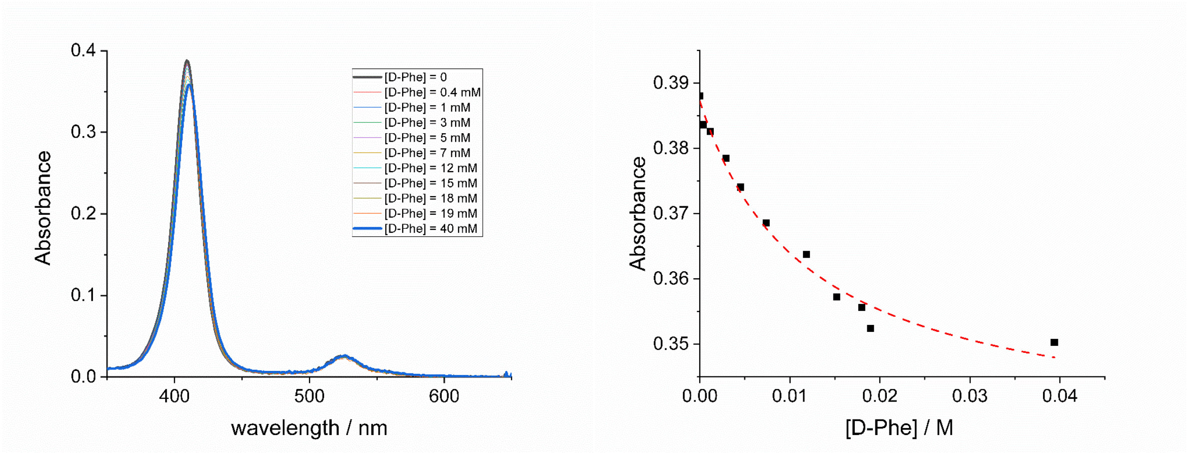

Figure S7. (left) UV/Vis absorption spectral changes for the progressive titration of $NiTPPS_4^{4-}$ (black line) with D-Phe up to a concentration of 40 mM (blue line). **(right)** Absorbance changes at the B-band of $NiTPPS_4^{4-}$ as function of the total concentration of added D-Phe. The red curve is the best fitting of the experimental data to eq. 6 (see Materials and methods). Experimental conditions: $[NiTPPS_4^{4-}]$ = 1.5 μM; [D-Phe] = 0 – 40 mM; acetate buffer 100 mM, pH = 4; T = 298 K; cell path length 1 cm.

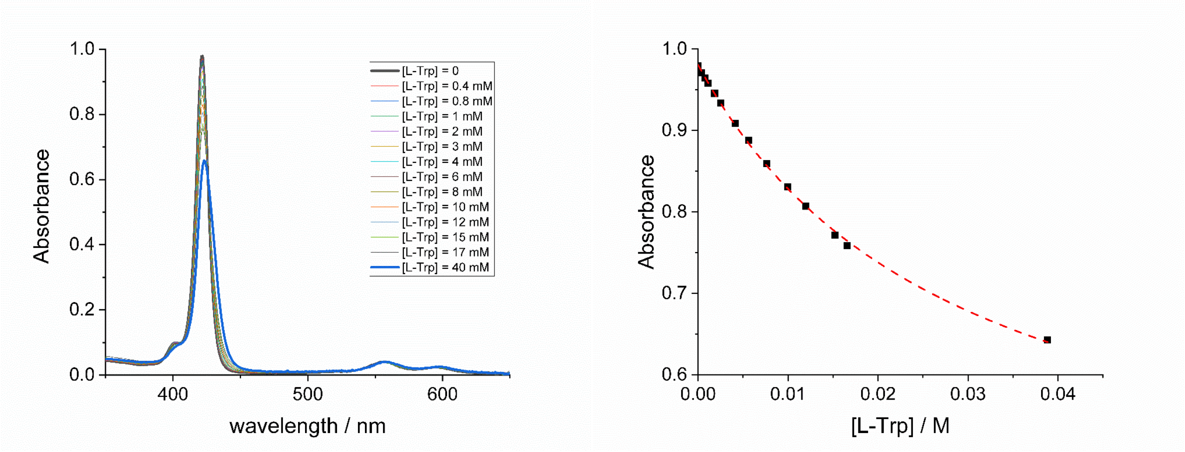

Figure S8. (left) UV/Vis absorption spectral changes for the progressive titration of $ZnTPPS_4^{4-}$ (black line) with L-Trp up to a concentration of 40 mM (blue line). **(right)** Absorbance changes at the B-band of $ZnTPPS_4^{4-}$ as function of the total concentration of added L-Trp. The red curve is the best fitting of the experimental data to eq. 6 (see Materials and methods). Experimental conditions: $[ZnTPPS_4^{4-}]$ = 1.5 μM; [L-Trp] = 0 – 40 mM; acetate buffer 100 mM, pH = 4; T = 298 K; cell path length 1 cm.

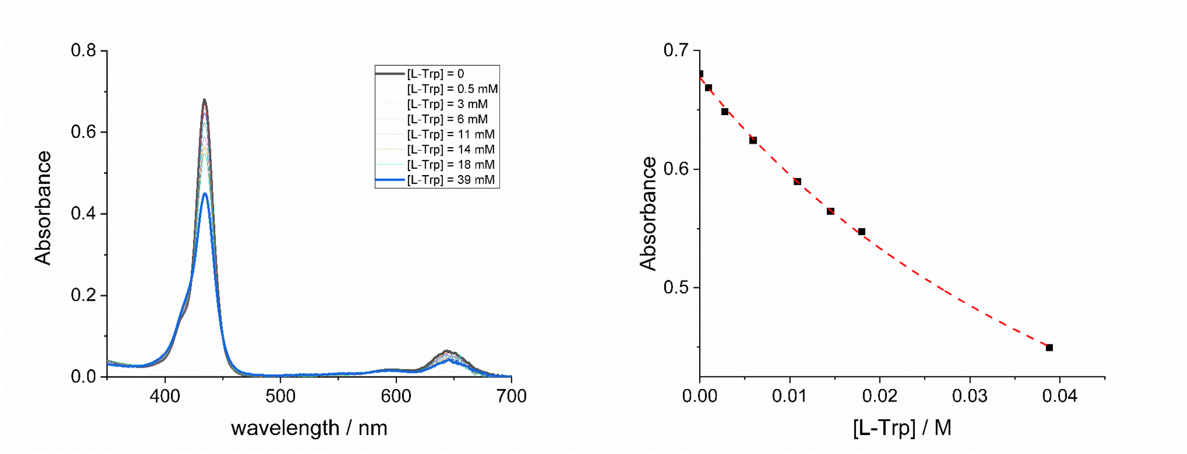

Figure S9. (left) UV/Vis absorption spectral changes for the progressive titration of $H_2TPPS_4^{2-}$ (black line) with L-Trp up to a concentration of 40 mM (blue line). **(right)** Absorbance changes at the B-band of $H_2TPPS_4^{2-}$ as function of the total concentration of added L-Trp. The red curve is the best fitting of the experimental data to eq. 6 (see Materials and methods). Experimental conditions: [$H_2TPPS_4^{2-}$] = 1.5 μM; [L-Trp] = 0 – 39 mM; acetate buffer 100 mM, pH = 4; T = 298 K; cell path length 1 cm.

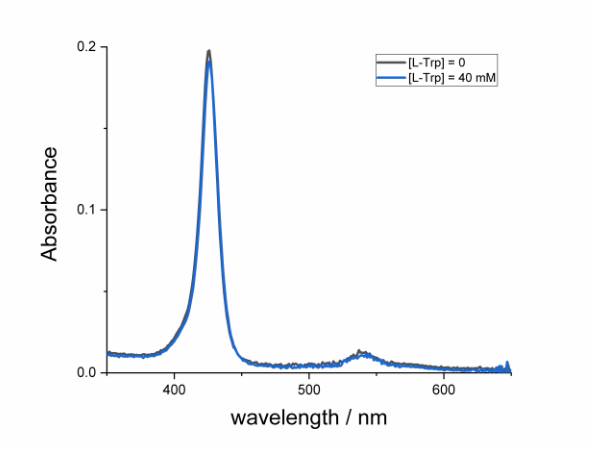

Figure S10. UV/Vis spectra of $CoTPPS_4^{4-}$ (black line) and in presence of a concentration of 40 mM of L-Trp (blue line). Experimental conditions: [$CoTPPS_4^{4-}$] = 1.5 μM; [L-Trp] = 40 mM; acetate buffer 100 mM, pH = 4; T = 298 K; cell path length 1 cm.

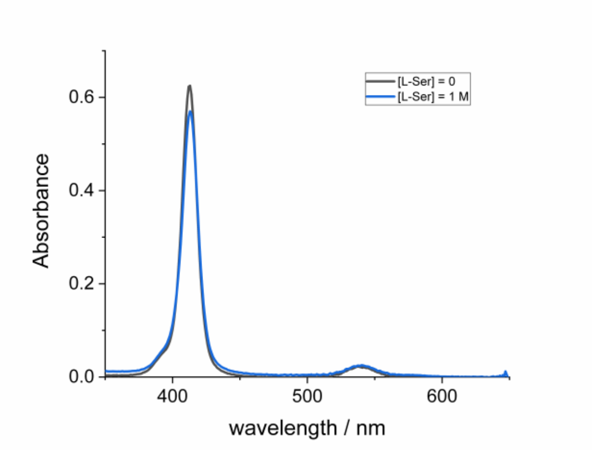

Figure S11. UV/Vis spectra of $CuTPPS_4^{4-}$ (black line) and in presence of a concentration of 1 M of L-Ser (blue line). Experimental conditions: [$CuTPPS_4^{4-}$] = 1.5 μM; [L-Ser] = 1 M; acetate buffer 100 mM, pH = 4; T = 298 K; cell path length 1 cm.
